# Cross-Cultural Adaptation and Measurement of Psychometric Properties of the Lithuanian Version of the Western Ontario Shoulder Instability Index (WOSI)

**DOI:** 10.3390/medicina60010117

**Published:** 2024-01-08

**Authors:** Kasparas Trukšnys, Aneta Bobin, Rokas Bobina, Simonas Utkus, Valentinas Uvarovas, Sigitas Ryliškis

**Affiliations:** 1 Centre of Family Medicine, Vilnius University Hospital Santaros Klinikos, LT-08661 Vilnius, Lithuania; 2 Faculty of Medicine, Vilnius University, LT-03101 Vilnius, Lithuania; bobin.aneta@gmail.com; 3 Clinic of Rheumatology, Orthopaedics Traumatology and Reconstructive Surgery, Faculty of Medicine, Vilnius University, LT-03101 Vilnius, Lithuania; rokasbo@outlook.com (R.B.); simonas.utkus@mf.stud.vu.lt (S.U.); valiusuvarovas@gmail.com (V.U.); ryliskis.s@gmail.com (S.R.); 4 Centre of Orthopaedics and Traumatology, Republican Vilnius University Hospital, LT-04130 Vilnius, Lithuania

**Keywords:** WOSI, shoulder instability, psychometric properties, validation, translation and cultural adaptation

## Abstract

*Background and Objectives*: The Western Ontario Shoulder Instability Index (WOSI) is a disease-specific self-administered questionnaire which is designed to measure health-related quality of life for patients with shoulder instability. The objective of this study was to translate and adapt the WOSI questionnaire for the Lithuanian-speaking population and investigate the psychometric properties of the Lithuanian version of the WOSI questionnaire (WOSI-LT): validity, reliability, and responsiveness. *Materials and Methods*: The WOSI scale was translated into Lithuanian using D. E. Beaton’s systematic and standardized guidelines for cross-cultural adaptation of patient-administered scales. Subsequently, the psychometric properties of the Lithuanian version of the scale (WOSI-LT) were investigated. The study involved 40 patients who reported shoulder instability and underwent surgical treatment. All patients completed the WOSI-LT, QuickDASH, and SF-12 scales. A subset of 10 patients was selected for the reproducibility and responsiveness evaluation. Based on the obtained data, the reliability, validity, and responsiveness of WOSI-LT were examined using statistical analysis methods. *Results*: The Lithuanian adaptation of the WOSI questionnaire exhibited a high degree of internal consistency, evidenced by a Cronbach’s alpha of 0.93. Its reproducibility was commendable with an intraclass correlation coefficient (ICC) value of 0.90. When assessing correlations, WOSI-LT demonstrated a stronger relationship with QuickDASH (r = 0.64) than with SF-12 (physical component score (PCS) 0.61, mental component score (MCS) 0.33). Six months post-operation, the responsiveness of the WOSI-LT was particularly notable, with a standardized response mean (SRM) of 0.91, the highest among the three scales. Furthermore, no floor or ceiling effects were identified in the scores of the Lithuanian WOSI. *Conclusions*: WOSI-LT is a valid, reliable, and responsive questionnaire that correlates excellently with the original English version of the scale. This scale can be used in Lithuanian medical institutions to assess the severity of patients’ shoulder instability and evaluate their progress during treatment.

## 1. Introduction

In clinical practice, assessing the impact of shoulder instability on a patient’s daily activities is important. Such evaluation is not only important when choosing appropriate treatment methods but also facilitates the monitoring of therapeutic outcomes. Instruments designed to evaluate the effects of shoulder instability in daily life can be categorized into general, limb-specific, joint-specific, and disease-specific questionnaires. The scales work most accurately when they are used for a narrower pathology, that is, disease-specific scales. Currently, the best-rated patient-specific scale for shoulder instability is the Western Ontario Shoulder Instability Index (WOSI) [1,2]. This disease-specific questionnaire is the only one created using a methodological system for developing quality of life scales [3]. The scale’s responsiveness to changes is particularly valued, making the WOSI very useful in tracking a patient’s shoulder instability during treatment. This scale holds significant importance to us, as highly experienced specialists have reached a consensus recommending the WOSI as the primary outcome measure for all studies addressing shoulder instability [4]. For these reasons, we determined that the Lithuanian population would greatly benefit from this tool designed to assess shoulder instability.

The WOSI questionnaire contains 21 items. The first domain, physical symptoms, has ten elements. Sports, recreation, and work (four items); lifestyle (four items); and emotions (three items) are the remaining domains. The patient scores each item on a visual analogue scale which indicates scores ranging from 0 to 100.

As far as we know, the WOSI has been translated and validated in 14 languages, which include Swedish, German, Japanese, Turkish, Arabic, Italian, Spanish, Danish, French, Hebrew, Dutch, Portuguese, Persian and Polish.

In Lithuania, to our knowledge, there are no validated scales suitable for assessing shoulder instability. Creating a new questionnaire for a small country is reasonable, as this work is very lengthy and requires significant financial and intellectual resources. Moreover, a newly created Lithuanian questionnaire could cause confusion and additional discussions in the literature, and it is unlikely that it would be better than previously created questionnaires. For these reasons, we decided to adopt a globally recognized tool for measuring shoulder instability for the Lithuanian population and create the Lithuanian version of the Western Ontario Shoulder Instability Index (WOSI-LT).

## 2. Materials and Methods

Guidelines developed by Guilemin et al. “Guidelines for the Process of Cross-Cultural Adaptation of Self-Report Measures” were used for cross-cultural validation and adaptation of the WOSI-LT [5].

Firstly, the original questionnaire was translated into Lithuanian by two Lithuanians for whom English was a second language. One of them had medical knowledge and knew about the purpose of the scale. The second translator had no medical degree and no knowledge of the purpose of the project.

Secondly, the translation of the first translator (T1) and the translation of the second translator (T2) were compared and in the presence of the third coordinating person medical doctor who had nothing to do with the research), a general translation of the scale was made (T1-2), which was acceptable for both translators.

Thirdly, the scale was translated back into English (BT1-2). It was completed by two translators (BT1, BT2), whose native language was English, and they both had no medical degree and no knowledge of the purpose of the project. This was to make sure that the translated version reflected the same item content as the original versions.

Fourthly, a committee of experts was assembled. It included an orthopedic trauma surgeon, a methodologist, a Lithuanian language specialist, and all the translators (forward and reverse translators) involved up to that point. All translations were looked over one more time and the final version of the WOSI-LT was created. The committee ensured scale’s semantic, idiomatic, experiential, and conceptual equivalence to the original version. The final version of the Lithuanian WOSI scale with reverse translations was sent to Sharon Griffin Laity, who is subject to the copyright of the original scale. She concluded that the questionnaire correlation with the original WOSI was good. Permission was given to start the analysis of the psychometric properties of the WOSI-LT.

Inclusion criteria: Traumatic instability of the shoulder (dislocations, fear of dislocation); all patients were selected for surgical treatment.

Exclusion criteria: (1) Psychiatric diseases, inability to speak Lithuanian, or inability to complete the questionnaire due to cognitive impairment. (2) Other diagnosis: an associated fracture of the scapula or humerus, shoulder arthritis, neurovascular involvement of the affected limb, inflammatory or neoplastic disorders, cervical radiculopathy, and thoracic outlet syndrome. (3) Other serious illnesses that can strongly influence the quality of life of a subject.

Patients: The evaluation of psychometric properties included 40 patients, with a mean age of 36.5 years (range 18–61). All patients were recruited in Republican Vilnius University Hospital. Informed consent was obtained from all subjects involved in the study. Subjects filled in WOSI-LT, SF-12, and QuickDASH questionnaires, which were given in Lithuanian (native) language. Later these scales were analyzed to examine the reliability, validity, and floor and ceiling effects of WOSI-LT. A total of 10 patients filled in the questionnaires 3 times. During the first orthopedic surgeon’s appointment, they filled in all the scales for the first time. After two weeks they filled the WOSI-LT scale again for test-retest evaluation. This period was chosen because 2 weeks lack time for changes to occur without surgical intervention and it is enough time for patients to forget their previous answers, therefore, the reproducibility of the scale could be evaluated. Patients also filled in all 3 scales again 6 months after surgery to evaluate the responsiveness of the scales.

Reliability: The reliability of assessment tools can be divided into two groups: the homogeneity (internal consistency) of a scale and the reproducibility (test-retest) of scores. Internal consistency was calculated using Cronbach’s alpha coefficient, using a 95% confidence interval. Cronbach’s alpha value of ≥0.7 is considered acceptable [6]. Reproducibility was evaluated by counting the intraclass correlation coefficient (ICC). ICC values between 0.5 and 0.75 indicate moderate reliability, values between 0.75 and 0.9 indicate good reliability and values greater than 0.90 indicate excellent reliability [7].

Validity: Validity is an index of how well a tool measures what it is supposed to measure. Validity is tested by comparing correlation with other questionnaires (or instruments) intended to measure the same outcome. To obtain the results we calculated the Pearson’s correlation coefficient. Pearson’s correlation coefficient is considered moderate from 0.5 to 0.7, strong from 0.7 to 0.9, excellent above 0.9 [8]. Overall WOSI-LT result was compared with QuickDASH and SF-12 physical and mental domains. Our hypothesis was that QuickDASH and SF-12 physical domain would correlate more strongly with WOSI-LT, while it would show little correlation with SF-12 mental domain.

Responsiveness: Responsiveness refers to the ability of a tool to measure change over time. We used a standardized response means for evaluating the responsiveness of the scale, the same measurement for responsiveness as A. Kirkley, S. Griffin, et al. used in the original WOSI version. [9] Standardized response mean is the mean change in score divided by the standard deviation of the change scores. Scale is considered to have good responsiveness when the standardized response mean is 0.8 and higher [10].

Floor and ceiling effects: Floor and ceiling effects are also considered important for the sensitivity analysis of the scale. It is the worst possible and the best possible values in the scale. Floor or ceiling effects are considered to be present if more than 15% of respondents achieve the lowest or highest possible score, respectively [11].

Data Analysis: Data processing and statistical analysis were performed using “Microsoft Excel” and “R Commander” version 2.8-0. All statistical parametric tests were performed after using the Shapiro–Wilk test to ensure that the data were normally distributed.

Ethics approval was obtained from the ethics committee in Republican Vilnius University Hospital (2020-12-15 Nr. 2R-5.5-4702).

## 3. Results

### 3.1. Translation and Cultural Adaptation

The translation and cultural adaptation process was completed in accordance with the procedure discussed above and no problems were encountered at this stage.

### 3.2. Patient Data

A total of 40 patients participated in the study to determine WOSI-LT construct validity and reliability (Table 1). A total of 72.5% (29/40) of subjects were male, while 27.5% (11/40) were female. The mean age of the patients was 36.5 years old. A total of 57.5% had a left shoulder instability, while 42.5% right one. All patients had recurrent dislocations and were selected for surgical treatment. A total of 10 patients were included in the reproducibility and responsiveness analysis.

### 3.3. Reliability

Reliability was tested by evaluating WOSI-LT internal consistency with Cronbach’s Alpha coefficient (Table 2). Cronbach’s Alpha was found to be 0.93. This value indicates that the scale has a high level of internal consistency. WOSI-LT’s four subscales also showed high-scale reliability. Intraclass correlation for the testing of reproducibility was fairly high not only for the full scale but also for the different WOSI-LT domains. Results show that the WOSI-LT scale and all subscales are stable over time.

### 3.4. Validity

The validity of the WOSI-LT questionnaire was evaluated by comparing results with other scales that are supposed to measure similar characteristics (Table 3). Pearson’s correlation coefficient between WOSI-LT and QuickDASH was 0.64, which was the highest correlation in this study. As expected, WOSI-LT showed a higher correlation with SF-12 physical component score (PCS) than SF-12 mental component score (MCS) (0.61, and 0.33, respectively).

### 3.5. Responsiveness

WOSI-LT showed the highest responsiveness of all the scales (Table 4). This means that WOSI-LT is sensitive to change over time. SF-12 mental score was the least responsive. Similar observations were seen in the original WOSI analysis.

### 3.6. Floor and Ceiling Effects

The analysis of the worst and best status values, which are considered important measures for the sensitivity analysis of the scales, showed no floor and ceiling effects (Table 5). In the Sports/Recreation/Work domain, a ceiling effect was observed in 2.5% of all subjects. In the Sports/Recreation/Work and Lifestyle domains, floor effect was observed in 2.5% of all subjects. In the postoperative WOSI-LT assessment, 10% of subjects reached the ceiling effect in the Sports/recreation/work, Lifestyle, and Emotions subscales.

## 4. Discussion

This study has demonstrated that the WOSI-LT had similar psychometric properties compared to the original English version of the WOSI. The WOSI-LT is a necessary tool that may be used to compare and interpret the data obtained from the Lithuanian population. Such data would allow us to further analyze the shoulder instability in international studies and meetings. The WOSI-LT was adapted following a systematic, standardized approach [5,12]. No difficulties were encountered in translating the questionnaire and the reverse translation was well comparable with the English version and fully approved by one of the WOSI authors. Patients who have filled in the WOSI-LT reported having no difficulty understanding questions and instructions.

We expected to receive a high Cronbach’s alpha coefficient, as the scale consists of 21 items (questions). The more items there are in the scale, the higher the Cronbach’s alpha value is expected [13]. Since researchers from all other countries adapting this questionnaire reported the coefficient, we were able to compare the results.

Cronbach’s Alpha coefficient, which indicates internal consistency compared to other cross-cultural adaptations, was found to be of the highest standard (0.93). Similar results were found in Swedish (0.89–0.95); Italian (0.93); German (0.92); Dutch (0.93–0.96); Turkish (0.91); Persian (0.93); Polish (0.94); Portuguese (0.97) versions [14,15,16,17,18,19,20,21]. Cronbach’s alpha coefficient was not assessed in the original WOSI, therefore, no comparison can be made between our version and the original version.

Reproducibility or test-retest analysis showed high WOSI-LT stability over time. ICC value was 0.90, which is considered good, and 0.78–0.89 variation for subscales [22]. We selected a two-week testing period, consistent with the original WOSI version. In comparison English version’s ICC value for the total WOSI was found to be 0.96, and it was found to be between 0.71 and 0.94 (2-week period) for the subscales. A wide range of results for the ICC was obtained in various countries: France: ICC 0.84, Japan: ICC 0.91, Italy: ICC at 3 days 0.95, ICC at 14 weeks 0.92, Arabic: ICC 0.96, Turkish: ICC 0.97, Polish: ICC 0.99 [15,18,21,23,24,25]. Our findings yielded a slightly lower ICC, which remains commendable. This deviation may be attributed to the constrained sample size of our participant group.

Construct validity of the Lithuanian WOSI was tested by comparing its results with QuickDASH and SF-12 questionnaires. We used Pearson’s correlation value to compare the results. They revealed a stronger association between the disease-specific WOSI-LT and the QuickDASH which measures similar parameters but is region-specific (r = 0.64). A weaker association was found between the WOSI-LT and the SF-12 which represents a less convergent construct: PCS (r = 0.61), MCS (r = 0.33). In the original research of WOSI, similar results were found. The WOSI total score strongly correlated with the DASH questionnaire (r = 0.77), weaker with the SF-12 Physical Component (r = 0.66), and weakest with the SF-12 Mental Component (r = 0.12). [9] A strong correlation with DASH was found in the Italian version (r = 0.79) and Polish version (r = 0.83) as well [15,21]. Our correlation was closer to Turkish (r = 0,67), French (r = 0.65), Japanese (r = 0.63), and Arabic (r = 0.62) versions of WOSI [18,23,24,25]. It is hard to compare the results of the Short Form Health Survey questionnaire because most of the countries did not distinguish the mental and physical factors of SF-36 or SF-12. To our knowledge, only Polish and Japanese studies obtained these results and Polish were very similar to our PCS (r = 0.77) and MCS (r = 0.35) [21].

When examining each questionnaire, it is very important to check for the absence of “floor and ceiling” effects. In the WOSI-LT scale, neither the floor nor the ceiling effect appeared (n = 40, 0–0%, respectively).

To evaluate the responsiveness of WOSI-LT, we utilized the standardized response mean (SRM). Of the eight preceding studies that assessed the responsiveness of their WOSI questionnaire, most indicated moderate to high scale responsiveness [9,14,15,21,23,25,26,27]. Our result, with an SRM of 0.91, demonstrated high scale responsiveness, closely mirroring the original WOSI version’s SRM of 0.93. The ability of a scale to detect changes over time is crucial. It offers a versatile tool for comparing the effectiveness of different treatment methods in enhancing an individual’s quality of life. WOSI is renowned for its exceptional ability to detect changes following surgical procedures. Among shoulder disease-specific questionnaires, WOSI boasts the highest responsiveness [28].

The study has several limitations, notably the sample size. We had 40 participants, whereas the recommended sample size for validation studies is 50 patients [29]. Ten subjects participated in test-retest and responsiveness measurement. The results obtained should be interpreted with caution, and the research must be continued to draw reliable conclusions regarding the responsiveness and repeatability of the tool. Furthermore, all our patients underwent surgical treatment, either through arthroscopy or the open Latarjet procedure. The scale should also be evaluated in patients treated conservatively.

## 5. Conclusions

Despite the limitations of the study, our data shows that the Lithuanian version of the shoulder joint instability questionnaire (WOSI-LT) is a reliable, valid patient-reported outcome measure for evaluating shoulder instability. Taking into account the limitations of the work related to the small group (n = 10), the conclusions related to the assessment of the responsiveness of the WOSI-LT require improvement. Research on the responsiveness of WOSI-LT requires continuation to provide reliable results. The Lithuanian version of the questionnaire demonstrated results similar to both the original and the WOSI scales translated and adapted for the native-speaking populations in other countries. Since it is the only shoulder instability specific questionnaire in Lithuania adapted and validated according to international guidelines, we recommend using it to assess the status and changes in patients suffering from shoulder instability during their treatment.

## Figures and Tables

**Table 1:** Subjects’ characteristics and clinical data.

Variables		Reliability and Validity Group	Responsiveness and Reproducibility Group
Age (mean)		36.5	31.4
Sex	Male	29	8
	Female	11	2
Injured side	Right	23	6
	Left	17	4
Repeatability		40/40	10/10
WOSI total score		1145.3 ± 392.9	
WOSI total score 2 weeks		1097.1 ± 405.6	
WOSI total score 6 months			289.4 ± 141.1
QuickDASH total score		40.8 ± 10.0	
SF-12 domains	Mental	43.5 ± 8.7	
	Physical	73.7 ± 8.1	

**Table 2:** Summary of reliability test of the WOSI-LT.

		ICC Test-Retest (*n* = 10)	Cronbach’s Alpha (*n* = 40)
WOSI total score		0.90	0.93
WOSI subscales	Physical symptoms	0.89	0.87
	Sport/recreation/work	0.82	0.75
	Lifestyle	0.84	0.82
	Emotions	0.78	0.80

**Table 3:** Correlation between the WOSI, the QuickDASH, and the SF-12.

Questionnaire	Pearson’s Correlation Coefficient	*p* Value
QuickDASH	0.64	*p* < 0.001
SF-12 PCS	0.61	*p* < 0.001
SF-12 MCS	0.33	*p* = 0.008

**Table 4:** The Standardized response mean of the tested outcome measures.

Outcome Measure	Standardized Response Mean
WOSI	0.91
QuickDASH	0.77
SF-12 PCS	0.51
SF-12 MCS	0.24

**Table 5:** The floor and ceiling effect of WOSI-LT.

		WOSI-LT Preoperative		WOSI-LT Postoperative	
		Floor Effect	Ceiling Effect	Floor Effect	Ceiling Effect
Full		0%	0%	0%	0%
Domains	Physical symptoms	0%	0%	0%	0%
	Sports/recreation/work	2.5%	2.5%	0%	10%
	Lifestyle	2.5%	0%	0%	10%
	Emotions	0%	0%	0%	10%

## Data Availability

The data presented in this study are available on request from the corresponding author. The data are not publicly available due to ethical restrictions.
